# Candicidal Effect of a Nanoemulsion Based on the Essential Oil of the Medicinal Plant *Haplopappus foliosus*: Role of Main Compounds in Yeast Inhibition

**DOI:** 10.3390/ph19050759

**Published:** 2026-05-12

**Authors:** Alejandro Madrid, Bastián Fuentes, Camila Araneda, Iván Montenegro, Nelson Caro, Valentina Silva, Evelyn Muñoz

**Affiliations:** 1 Laboratorio de Productos Naturales y Síntesis Orgánica (LPNSO), Facultad de Ciencias Naturales y Exactas, Universidad de Playa Ancha, Leopoldo Carvallo 270, Valparaíso 2360000, Chile; silvapedrerosv@gmail.com; 2 Doctorado en Ciencias Mención Biofísica y Biología Computacional, Universidad de Valparaíso, Valparaíso 2360103, Chile; bastian.fuentes@uv.cl; 3 Center of Interdisciplinary Biomedical and Engineering Research for Health (MEDING), Escuela de Obstetricia y Puericultura, Facultad de Medicina, Universidad de Valparaíso, Angamos 655, Reñaca, Viña del Mar 2520000, Chile; ivan.montenegro@uv.cl; 4 Instituto de Química, Pontificia Universidad Católica de Valparaíso, Valparaíso 2360000, Chile; camila.araneda.v@mail.pucv.cl; 5 Centro de Investigación Austral Biotech, Facultad de Ciencias, Universidad Santo Tomás, Avda. Ejercito 146, Santiago 8320000, Chile; nelsoncarofu@santotomas.cl; 6 Departamento de Química Orgánica, Facultad de Ciencias Químicas, Universidad de Concepción, Concepción 4070386, Chile; emunoznu@udec.cl

**Keywords:** Bailahuén, *Candida albicans*, yeast, natural, phytomedicine, *α*-bisabolol

## Abstract

**Background/Objectives**: The increasing resistance of *Candida* species to conventional antifungals, particularly azoles, poses a critical public health challenge due to high mortality rates associated with candidemia. This study aimed to describe the chemical composition of the essential oil from *Haplopappus foliosus* (EO-BAI) and evaluate its antifungal properties, along with its nanoemulsion (NE-BAI) and major constituents, against a panel of clinical *Candida* isolates. **Methods**: EO-BAI was extracted via steam distillation and analyzed using GC-MS. A nanoemulsion was synthesized through ultrasonic emulsification and characterized by DLS and microscopy (SEM/STEM). Antifungal activity (MIC/MFC) was determined following CLSI M27-A3 guidelines. Time–kill kinetic studies were conducted on *C. albicans*, and an in silico approach was used to describe interactions with Als9-2 and CYP51 targets. **Results**: The EO-BAI profile was dominated by terpinen-4-ol (27.27%) and *α*-bisabolol (10.40%). The NE-BAI exhibited a droplet size of approximately 22 nm with an encapsulation efficiency of 88.72%. Among the tested samples, *α*-bisabolol emerged as the core bioactive driver (MIC = 16 µg/mL against *C. albicans*). While NE-BAI showed reduced initial activity at 24 h, it demonstrated enhanced efficacy by 48 h, matching fluconazole’s potency and maintaining a definitive fungicidal effect. Docking analysis confirmed that *α*-bisabolol establishes stabilizing interactions with key virulence and membrane homeostasis targets. **Conclusions**: The NE-BAI provides a sustained delivery of its bioactive terpenes, preserving their fungicidal nature and positioning them as robust therapeutic alternatives to conventional treatments.

## 1. Introduction

*Candida* species are opportunistic pathogens that typically function as common components of the human microbiota, however, host alterations such as glucocorticoid use, immunosuppression, or uncontrolled diabetes can lead to *Candida* overgrowth [1]. Clinical manifestations range from local oral, esophageal, or vaginal mucocutaneous infections to severe cases of sepsis [2], among which candidemia represents a significant health challenge due to its high mortality rate (25–50%) [3]. Among the different *Candida* species, *Candida albicans* remains the most frequent cause of infection, accounting for nearly 50% of isolates in various reports [1,4]; furthermore, this species has demonstrated resistance to several antifungals, especially azoles, which were traditionally the most common form of control [2]. Therapeutic resistance of *C. albicans* to azoles, such as that reported for fluconazole is driven by the activation of efflux pumps that expel the drug from the cell, alterations or overexpression of genes that prevent azole binding, and the development of alternative metabolic pathways [5,6]. In this scenario of increasing resistance, even drugs such as amphotericin B may see their effectiveness reduced, since the pathogenicity of *C. albicans* is reinforced by its ability to form biofilms, which protect the yeast from antifungal agents [7]. In addition, recent epidemiological shifts have led to an increase in fungal co-infections and a higher prevalence of non-*albicans* species with intrinsic resistance [8].

Given this complicated scenario, there is a need for new antifungal agents capable of controlling *Candida* species. The literature shows that essential oils (EOs) are preferred due to their natural origin, and that the presence of different active ingredients capable of controlling microorganisms through different mechanisms of action hinders the development of resistance [9]. In this context, the *Elettaria cardamomum* (cardamom) EO stands out, as it has demonstrated potent anti-biofilm activity against clinical *Candida* isolates on polystyrene surfaces [10], in addition to cardamom, similar antifungal and antibiofilm properties have been documented in other plant resources, notably the *Cymbopogon nardus* EO [11], various species of the Lamiaceae family [12], and several Colombian oils characterized by a high sesquiterpene content [13]. Within this scope of botanical exploration, the genus *Haplopappus* is of particular interest, as its species have exhibited biological activity against a wide range of fungi, however, although extracts, infusions, and resinous exudates from this genus have been evaluated against *Candida* spp., the results reported to date have not been conclusive [14]. It should be noted that, despite this biotechnological potential, the study of its EO remains unexplored, this phenomenon may be influenced by the fact that these natural products suffer from limitations in their application due to their poor stability and solubility in water, which limits their therapeutic action [15]. However, new technologies allow for improvements in stability, such as the use of nanoemulsions, whose small particle size enhances the absorption of drugs through many routes and also increases their bioavailability [16].

In this regard, a literature review of the chemical composition of *Haplopappus foliosus* (Bailahuén) EO suggests a profile dominated by metabolites such as limonene, terpinen-4-ol, *p*-cymene, and caryophyllene [17,18]. This chemical characterization is of significant interest, as it aligns with the compositional profiles described by Hou and Huang (2024) for EOs with high potential for treating *C. albicans* infections, further supporting the hypothesis of its therapeutic efficacy [9]. Given this background, this research sought to describe the composition of *H. foliosus* EO and evaluate the antifungal properties of the oil (EO-BAI), its nanoemulsion (NE-BAI), and its major components against a panel of *Candida* species. Furthermore, an in-depth study was conducted on *C. albicans*—selected due to its clinical prominence as the leading cause of candidiasis worldwide and its high incidence in systemic infections. This specific focus aimed to determine time–kill kinetics and employ an in silico approach to describe the interactions of the bioactive compounds with the adhesive N-terminal domain of Als9-2 and the enzyme sterol 14α-demethylase (CYP51), targeting key mechanisms of fungal virulence and membrane homeostasis.

## 2. Results and Discussion

### 2.1. Oil Composition Analysis

Clevenger extraction of the EO-BAI yielded 0.06 (*v*/*w*) and a total of 56 compounds were identified in this oil. The results of the analysis of the chemical composition of the EO-BAI obtained through gas chromatography–mass spectroscopy (GC-MS) are shown in Table 1.

The chemical composition of EO-BAI is characterized by a marked predominance of oxygenated terpenes, with terpinen-4-ol (27.27%), *α*-bisabolol (10.40%), and spathulenol (6.70%) emerging as the primary constituents. This high concentration of terpenic alcohols is biologically relevant, as these compounds possess a greater capacity for disrupting the lipid membranes of pathogens compared to their hydrocarbon precursors. While many essential oils from the same botanical family are typically dominated by hydrocarbon monoterpenes like limonene or pinene, the EO-BAI profile shows a biosynthetic pathway more oriented toward oxygenated derivatives. This chemical signature is comparable to that reported for *Melaleuca alternifolia* (Tea Tree) EO, also rich in terpinen-4-ol [19], suggesting that EO-BAI could possess significant anti-*Candida* potential. It has been established that the synergistic action between terpinen-4-ol and oxygenated sesquiterpenes often enhances inhibitory activity against *Candida* sp. by altering membrane permeability and inhibiting germ tube formation [20,21,22,23].

Significant quantitative and qualitative variations are observed when contrasting these findings with previous reports for *H. foliosus*. Specifically, Urzúa et al. (2010) identified limonene (28.0%) and *epi*-bicyclosesquiphellandrene (9.84%) as the major components [18], compounds that were absent in our sample. Furthermore, although terpinen-4-ol was identified in both studies, its relative abundance in the present work (27.27%) significantly exceeds the 6.36% previously reported. These differences may be attributed to environmental factors inherent to the xerophytic coastal biome and the phytosanitary state of the plant. According to Villagra et al. (2021), the presence of terpinen-4-ol and other monoterpenes like *p*-cymene and *γ*-terpinene is associated with healthy apical branches, whereas insect-induced parasitism triggers an increase in limonene [17]. Moreover, the presence of *α*-bisabolol in this EO differs from what has been documented in previous research on extracts or resinous exudates of *H. foliosus* [24,25,26], where this oxygenated sesquiterpene was not identified. This chemical variability underscores the influence of biotic and abiotic conditions on the terpenoid biosynthetic pathways for this species and highlights the unique chemical profile of the oil evaluated in this study [27,28].

### 2.2. Nanoemulsion of Essential Oil

#### 2.2.1. Characterization

The physicochemical properties of NE-BAI, including DLS droplet size analysis, are presented in Table 2. A vehicle nanoemulsion, in which EO-BAI was replaced by distilled water, served as a control to assess the stability of the nanoemulsion matrix.

The physicochemical characterization of the nanoformulations revealed a pH of 6.90 for the Tween 80-based nanoemulsion and 6.88 for the oil-free control, both within the expected range for standard conditions [29]. Regarding morphology, the formulation exhibited a particle size of approximately 22 nm, meeting the criteria for a nanoemulsion [30]. This size is comparable to the 20–30 nm range described for similar essential oil-loaded systems processed by sonication [31], whereas systems stabilized by surfactants with high hydrophilic-lipophilic balance values typically exhibit slightly larger particles [32,33]. Furthermore, the formulation achieved a PDI of 0.583, falling within the optimal range (0.05 to 0.7) and ensuring predictable compound release [29,34].

In terms of physical stability, the nanoemulsion showed a ZP of −3.47 mV. While ZP values greater than ±30 mV are typically associated with electrostatic stability [35], the absence of flocculation in this system—despite low electrostatic repulsion—confirms that stability is primarily driven by the steric hindrance provided by Tween 80 and Pluronic F108 [36]. This stability directly contributes to an EE of 88.72% for the EO-BAI, demonstrating the effectiveness of these surfactants [37]. Furthermore, the EE remains stable because the formulation avoids high ionic strength conditions; at low ionic concentrations, the electrostatic repulsion between droplets is sufficient to resist aggregation and flocculation [38]. Conversely, it has been reported that high ionic concentrations can compromise the stability of such nanoemulsions, leading to potential degradation [39].

#### 2.2.2. Morphology

**Figure 1:**
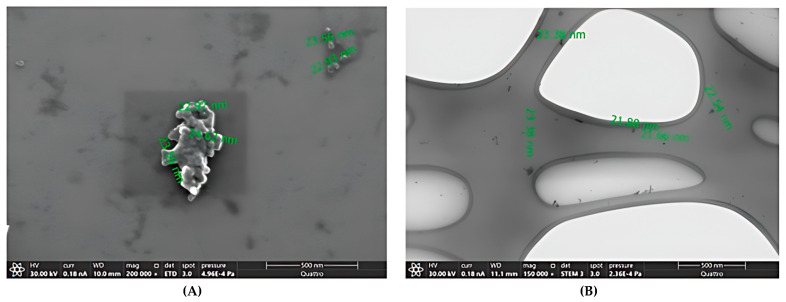
(**A**) SEM image of nanoemulsion formulation, (**B**) STEM image of nanoemulsion.

To complement the results obtained in the DLS measurement, an SEM/TEM analysis was performed, Figure 1 shows the morphology of the nanodroplets.

The mean particle size, determined by dynamic light scattering (DLS), was 21.80 ± 5.1 nm (Table 2), which is consistent with measurements obtained by scanning electron microscopy (SEM), which showed nanoemulsion droplets with a size ranging from 21 to 25 nm, and by scanning transmission electron microscopy (STEM), which indicated sizes between 21 and 24 nm (Figure 1). These DLS results are validated by confirming that the measurements represent individual particles rather than agglomerates. Nevertheless, to comply with regulatory standards (ASTM E3247-20 and FDA Guidance) that highlight the inherent limitations of DLS in distinguishing between individual particles and aggregates [37], we recommend incorporating advanced microscopy techniques as a complementary validation method.

### 2.3. Anti-Candida Activity

The susceptibility of different *Candida* strains to NE-BAI, EO-BAI, and their major constituents was evaluated. Specifically, pure compounds representing more than 10% of the total essential oil composition—*α*-bisabolol (10.40%) and terpinen-4-ol (27.27%)—were selected for MIC and MFC assays. The results, along with those for the nanoemulsion and reference controls, are summarized in Table 3.

The antifungal profile of the treatments remained consistent across the evaluated *Candida* sp., revealing a clear time-dependent efficacy. At 24 h, EO-BAI exhibited moderate to high antifungal activity, with MIC_80_ values of 32 µg/mL for *C. albicans* and *C. tropicalis*, and a remarkable potency against *C. lusitaniae* (MIC_80_ = 8 µg/mL). In contrast, the NE-BAI showed markedly reduced activity at this initial time point (MIC_80_ > 256 µg/mL) for most strains (refer to Table S1 in the Supplementary Material). This initial lag in growth inhibition is characteristic of nanoemulsion systems, where the bioactive terpenes are sequestered within the oil droplets, preventing immediate contact with the fungal cells.

However, the therapeutic window improved significantly over time. By 48 h, the NE-BAI demonstrated enhanced efficacy, reducing the MIC_80_ to 64 µg/mL for *C. albicans* and *C. tropicalis* (Table 3). This transition from an inactive state at 24 h to a potent inhibitory state at 48 h suggests a delayed-release kinetic mechanism, consistent with nanoformulations that protect volatile compounds from degradation while ensuring a sustained delivery [40,41]. From a methodological perspective, these results highlight that MIC-based endpoints alone are sometimes decoupled from true viability, particularly in yeasts displaying “trailing” or tolerance phenomena [42]. A critical aspect of these results is the MFC/MIC ratio, which was consistently ≤4 for EO-BAI, NE-BAI, and *α*-bisabolol across nearly all susceptible strains. According to established microbiological criteria, a ratio within this range indicates a definitive fungicidal effect rather than a merely fungistatic one [43]. This suggests that the nanoemulsion does not compromise the lethal mechanism of the EO; instead, it facilitates the delivery of the active principles to the fungal membrane, leading to irreversible cell death. Among all tested samples, *α*-bisabolol emerged as the core bioactive driver, demonstrating superior antifungal efficacy across the entire spectrum of strains, consistent with previous reports of its potent activity against *Candida* sp. and its interaction with fungal cytochrome P450 involved in ergosterol biosynthesis [44,45]. The fact that *α*-bisabolol and the NE-BAI maintained low ratios even in *C. tropicalis* and *C. lusitaniae* highlights their potential as robust fungicidal agents. Finally, the lack of activity in the nanoemulsion vehicle and DMSO controls (MIC_80_ > 256 µg/mL) confirms that the observed effects are strictly derived from the phytochemical constituents [43].

When comparing these results with reference antifungal drugs, it was observed that the EO-BAI and its major component, *α*-bisabolol, exhibit comparable or even superior potency to fluconazole against critical strains. For instance, *α*-bisabolol four-fold higher potency than fluconazole against *C. albicans* (MIC values of 16 vs. 64 µg/mL). Although itraconazole maintained the lowest MIC values in absolute terms, the clinical relevance of the NE-BAI lies in its mode of action. The comparison with clotrimazole further emphasizes the clinical potential of the developed treatments. Despite being a gold-standard topical antifungal, clotrimazole showed no activity against *C. lusitaniae* and *C. tropicalis* (MIC > 256 µg/mL), whereas NE-BAI and *α*-bisabolol maintained high susceptibility in these strains. Furthermore, while clotrimazole matched the MIC of NE-BAI in *C. albicans* (64 µg/mL), it was twice as less potent as the nanoemulsion against *C. glabrata* (256 vs. 128 µg/mL). While azoles often act as fungistatic agents, potentially leading to fungal persistence, NE-BAI successfully preserves the fungicidal profile of the original EO (R ≤ 4) across the entire evaluated spectrum. The ability of NE-BAI to induce irreversible cell death, combined with its capacity to match fluconazole’s potency at 48 h, demonstrates that encapsulation not only stabilizes the volatile constituents of the oil but also positions them as robust therapeutic alternatives to conventional treatments, particularly in cases where azole tolerance limits clinical success.

While the MIC and MFC assays provided a broad overview of susceptibility across various species, time–kill kinetic studies were focused exclusively on *C. albicans*. This selection is justified by its clinical prominence as the leading cause of candidiasis worldwide and its complex virulence factors, which make it the primary target for new antifungal drug delivery systems [45]. This integrated approach allowed us to clarify potential misinterpretations from static endpoints, revealing that while the EO provides a rapid initial hit, its effect is non-sustained compared to the persistent suppression achieved by *α*-bisabolol [46,47].

The transition of NE-BAI from an initial lag phase at 24 h to a definitive fungicidal effect at 48 h suggests a cumulative cellular damage mechanism [48]. The sustained release of the essential oil (EO-BAI) from the nanoemulsion ensures a continuous exposure of the yeast to its bioactive terpenes [32,49]. This persistent delivery may trigger intracellular oxidative stress through the generation of Reactive Oxygen Species (ROS), leading to mitochondrial dysfunction and the induction of apoptosis-like cell death [50,51]. Unlike the free oil, which exerts a rapid but transient impact due to its high volatility, the nanoformulation maintains the necessary oxidative pressure to overcome fungal tolerance mechanisms, resulting in irreversible cell death [48,49]. These mechanistic insights provide the theoretical basis for the dynamic lethal effects observed in the subsequent time–kill analysis.

### 2.4. Time–Kill Kinetics

**Figure 2:**
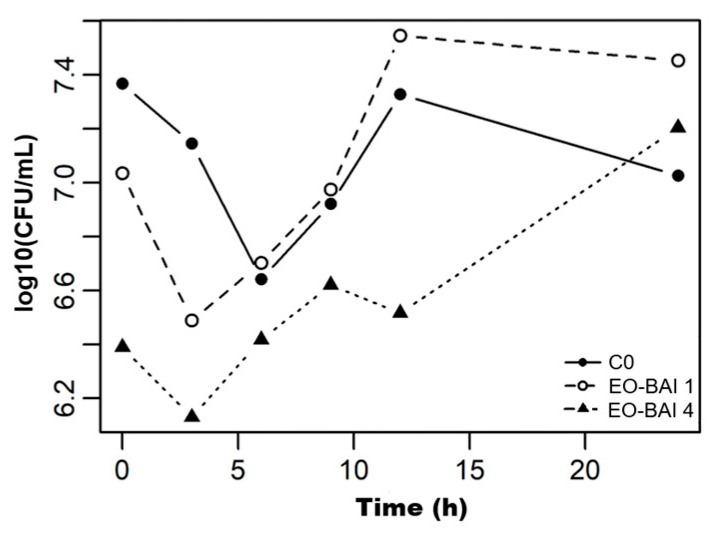
Time–kill kinetics of *C. albicans* exposed to EO-BAI. Treatments correspond to 1×MIC (EO-BAI 1), 4×MIC (EO-BAI 4). C0: Untreated growth control. Data are expressed as log_10_ CFU/mL.

**Figure 3:**
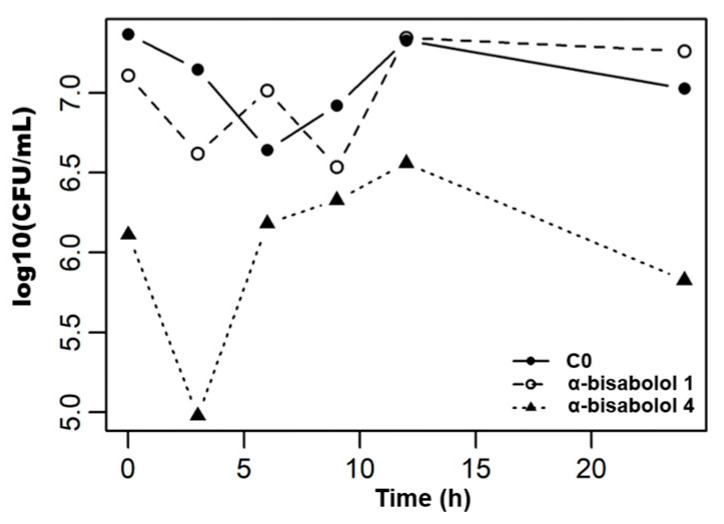
Time–kill kinetics of *C. albicans* exposed to *α*-bisabolol. Treatments correspond to 1×MIC (*α*-bisabolol 1), 4×MIC (*α*-bisabolol 4). C0: Untreated growth control. Data are expressed as log_10_ CFU/mL.

The dynamics of cell death in response to *C. albicans* are presented in Table 4 and illustrated in Figure 2 and Figure 3.

The time–kill analysis revealed distinct pharmacodynamic profiles among the tested agents. The untreated control (C0) and the VC exhibited a similar biphasic trajectory, characterized by an initial slight decrease at 6 h (independent of antifungal exposure), followed by recovery to approximately 7.0 log_10_ CFU/mL at 24 h. This pattern indicated a basal adaptation phase of the inoculum associated with the transition from solid Sabouraud Dextrose Agar (SDA) to liquid RPMI 1640 medium.

For the free EO-BAI, neither 1×MIC nor 4×MIC concentrations were able to prevent population recovery by the end of the experiment. While the 4×MIC exposure maintained the culture below the control levels for the first 12 h, it did not achieve a 3 as log_10_ reduction at 24 h, subsequently increasing to 7.20 as log_10_ CFU/mL. This recovery indicates a fungistatic effect under these specific experimental conditions, where the high volatility of the free terpenes might limit their long-term efficacy in the open-system of the time-kill assay.

In contrast, *α*-bisabolol at 4×MIC exhibited the most pronounced early activity, achieving a reduction from 6.11 to 4.98 log_10_ CFU/mL within the first 3 h (exceeding a 1-log decrease). Despite a partial recovery observed between 6 and 12 h, this treatment was the only one to maintain a net reduction by 24 h (5.83 log_10_ CFU/mL) relative to its baseline and the growth control. These results highlight the significant role of *α*-bisabolol in the antifungal profile of the EO-BAI. Although it is the second most abundant component (10.40%), it stands out as a key bioactive constituent, particularly since the major component, terpinen-4-ol (27.27%), was found to be inactive in preliminary MIC and MFC assays. Finally, the NE-BAI (1×MIC and 4×MIC) exhibited limited early activity during the first 24 h, with growth trajectories closely resembling the control (C0). This behavior is consistent with the delayed-release kinetic mechanism suggested by the 48 h MIC results. The sequestration of the active principles within the oil droplets likely shifts the maximum antifungal activity beyond the 24 h window, as previously reported for nanoemulsified EOs designed for sustained delivery [49,52]. Consequently, the NE-BAI should be interpreted not simply as “less active” than the free oil, but as a formulation with a temporally displaced window of action, where encapsulation shifts the balance between early and late antifungal effects.

### 2.5. Molecular Docking Analysis

The molecular docking results of the major constituents of EO-BAI against the N-terminal domain of Als9-2 from *C. albicans* (2Y7L) and sterol 14α-demethylase (CYP51, 5TZ1) are summarized in Table 5.

Overall, the positive controls exhibited the most favorable binding affinities, with itraconazole showing the lowest binding energies (−9.96 and −10.51 kcal/mol for 2Y7L and 5TZ1, respectively), followed by fluconazole (−7.97 and −6.91 kcal/mol). Clotrimazole also showed high affinity toward 5TZ1 (−9.01 kcal/mol), which is consistent with the well-established antifungal mechanism of azoles involving CYP51 inhibition and disruption of ergosterol biosynthesis [53,54,55].

Among the terpenoid constituents, *α*-bisabolol showed the best docking performance against both targets, with binding energies of −5.98 kcal/mol for 2Y7L and −6.01 kcal/mol for 5TZ1, outperforming terpinen-4-ol (−4.56 and −4.28 kcal/mol, respectively). Although these affinities were lower than those of the reference antifungal drugs, they suggest that selected constituents of EO-BAI may contribute to its overall antifungal activity through multitarget effects or synergistic interactions [56,57]. These results are particularly relevant considering the biological role of the targets studied. The 2Y7L receptor corresponds to the N-terminal adhesive domain of Als9-2, a member of the ALS family involved in host adhesion, colonization, and biofilm development [1,6]. By contrast, 5TZ1 represents sterol 14α-demethylase (CYP51), a key enzyme in ergosterol biosynthesis and one of the major molecular targets of azole antifungal agents [54,58,59]. Accordingly, both receptors provide relevant models for evaluating compounds with the potential to interfere with both virulence-related mechanisms and membrane homeostasis in *C. albicans*. In this context, *α*-bisabolol emerged as a key candidate, establishing significant stabilizing interactions with both targets. Regarding its binding mode in 2Y7L, it interacted with Phe225, Tyr23, Trp294, Ser170, Pro29, Val22, Leu179, and Val19, including a hydrogen bond with Ser170 (1.85 Å). In 5TZ1, it interacted with Tyr118, Leu121, Tyr132, Leu376, Phe380, Pro230, Phe233, and Met508, forming a hydrogen bond with Met508 (1.87 Å). These findings are consistent with recent evidence showing that *α*-bisabolol reduces ergosterol production in *C. albicans* and exhibits in silico affinity toward 14α-demethylase, supporting its potential role in disrupting ergosterol biosynthesis [45,60]. In contrast, terpinen-4-ol showed predominantly hydrophobic interactions with both receptors and lower relative binding affinity, suggesting a possible complementary contribution within the terpenoid profile of the EO-BAI [56]. Among the azoles, fluconazole and itraconazole displayed extensive interaction networks consistent with their well-established pharmacological profiles, whereas clotrimazole was particularly notable against 5TZ1, where it formed favorable interactions with key residues such as Tyr132 and Lys143, both of which are relevant for ligand recognition within the CYP51 binding pocket [53,61,62].

**Figure 4:**
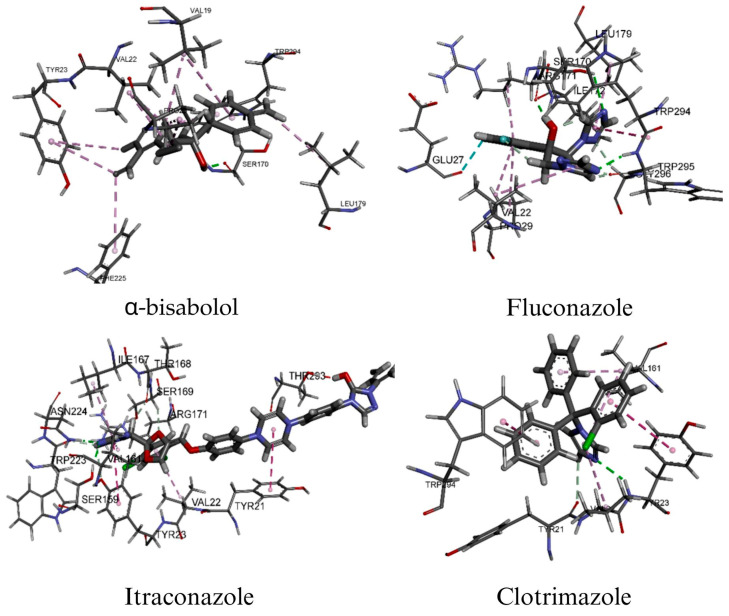
Three-dimensional binding modes of *α*-bisabolol and reference antifungal drugs within the active sites of Als9-2 (PDB: 2Y7L).

**Figure 5:**
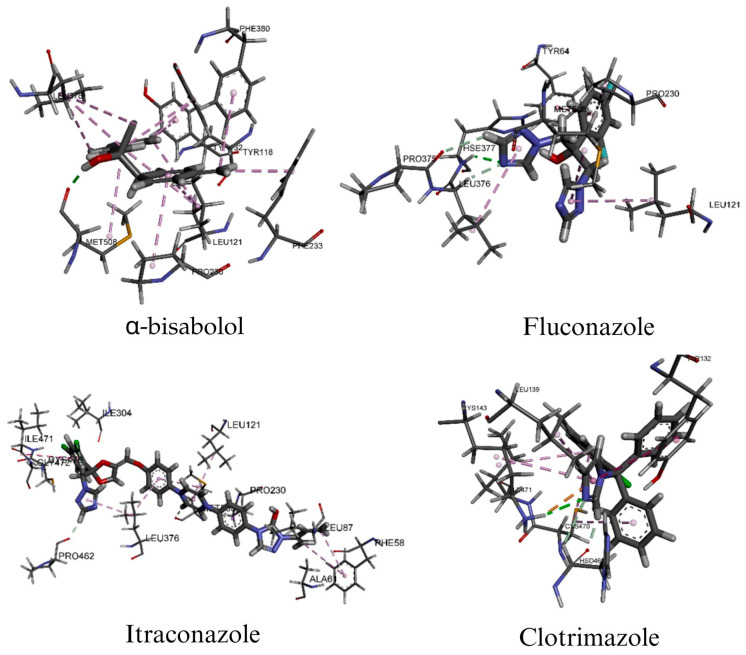
Three-dimensional binding modes of *α*-bisabolol and reference antifungal drugs within the active sites of CYP51 (PDB: 5TZ1).

Taken together, these findings indicate that the constituents of EO-BAI exhibit distinct molecular recognition patterns toward key *C. albicans* targets. As expected, the reference azoles showed the most favorable binding energies; however, *α*-bisabolol emerged as the most relevant docked constituent among the terpenoid compounds evaluated due to its comparatively higher affinity and the presence of stabilizing interactions with both targets (Figure 4 and Figure 5).

In combination with experimental evidence demonstrating its ability to reduce ergosterol biosynthesis and inhibit adhesion, yeast-to-hypha transition, and biofilm development, these data support the hypothesis that *α*-bisabolol may contribute significantly to the antifungal activity of the EO-BAI through a dual mechanism involving virulence modulation and disruption of membrane integrity [45,57]. Nevertheless, given that molecular docking is a target-restricted theoretical approach, these findings should be interpreted as supportive evidence and not as a replacement for experimental biological validation [56,63].

## 3. Materials and Methods

### 3.1. Pathogens

The antifungal activity of EO-BAI and its primary constituents was evaluated against a panel of four clinical isolates: *C. albicans* 10935, *C. glabrata* 10912, *C. lusitaniae* 2305, and *C. tropicalis* 9841. These strains were obtained from the fungal culture collection of the Biomedical Research Center (CIB), School of Medicine, University of Valparaíso, Chile. The isolates were maintained in SDB media supplemented with glycerol and stored at −80 °C according to established protocols [64]. Prior to testing, each isolate was subcultured to ensure purity and viability.

### 3.2. Plant Material

Plant samples were collected from Quilimarí, Coquimbo Region, Central Chile (S: 32.1176°, W: −71.4757°) during the spring in October 2025. Botanical identification and authentication were verified by Mr. Patricio Novoa, and a voucher specimen (BAI-1025) was deposited at the Natural Products and Organic Synthesis Laboratory of Universidad de Playa Ancha, Valparaíso, Chile.

### 3.3. Preparation of Essential Oil

The EO-BAI was extracted from the fresh aerial parts of *H. foliosus* (500 g). Prior to processing, the plant material was washed with distilled water to remove surface contaminants and air-dried at room temperature. Subsequently, the samples were ground in a knife mill. Extraction was performed via steam distillation using a Clevenger-type apparatus for 4 h [65]. The obtained hydrolate underwent liquid–liquid partition in a separatory funnel; the resulting EO were then dried over anhydrous sodium sulfate and stored in amber glass vials at −4 °C until further chemical and biological testing.

### 3.4. Chemical Analysis

The chemical composition of the EO-BAI was determined using a GC-MS/MS system (GC: Trace 1300; MS: TSQ8000Evo, Thermo Fisher Scientific, Waltham, MA, USA). The samples were diluted with dichloromethane, and 1 μL was injected into a splitless injector (250 °C). The system operated in electron ionization (EI) mode at 70 eV, with a transfer line temperature of 200 °C. Helium was used as the carrier gas at a flow rate of 1.2 mL/min. Separation was achieved using an Rtx-5 ms capillary column (60 m × 0.25 mm i.d., 0.25 μm film thickness). The oven temperature program started at 40 °C (held for 5 min) and increased to 300 °C (held for 5 min) at a rate of 5 °C/min. The identification of the EO-BAI components was performed by comparing their mass spectra with the NIST21 library (acceptance criterion: match value > 800) [66]. Identification was further confirmed by comparing the calculated retention indices (RI) with literature data.

### 3.5. Fabrication of EO-BAI-Loaded Nanoemulsion

The EO-BAI nanoemulsion was synthesized via ultrasonic emulsification, adapting a previously reported protocol [67]. Separate heating of the aqueous phase (distilled water containing Pluronic^®^ F108 (Sigma-Aldrich, St. Louis, Mo, USA)) and the organic phase (olive oil with Tween^®^ 80 (Sigma-Aldrich, St. Louis, Mo, USA)) was conducted at 60 °C. The EO-BAI was integrated into the oily carrier to reach a target concentration of 1000 ppm. Upon combining both phases, the coarse emulsion was pre-mixed in an ultrasonic bath for 10 min at 65 °C. High-energy emulsification was subsequently achieved using a Vibra-Cell VCX130 Ultrasonic Processor (Sonics & Materials Inc., Newtown, CT, USA) featuring a 1/4” probe. This process was carried out over 6 min at 90% amplitude, employing a pulse regime of 50 s on and 20 s off. For comparative purposes, a control (blank) nanoemulsion was synthesized following the same parameters substituting the EO-BAI with deionized water. The mean particle size (Z-average) and zeta potential were determined via Dynamic Light Scattering (DLS) and Phase Analysis Light Scattering (PALS), respectively, using a Zetasizer Nano ZS system (Malvern Instruments, Malvern, UK) [65]. Prior to analysis, samples were diluted 200-fold with deionized water. All formulations were stored at room temperature until further evaluation.

### 3.6. Antifungal Susceptibility Assays

The MIC and MFC were determined using the broth microdilution method according to the Clinical and Laboratory Standards Institute (CLSI) M27-A3 guidelines [58] with minor modifications.

#### 3.6.1. Inoculum Preparation

Clinical isolates were subcultured on SDA and incubated at 37 °C for 24–72 h. Fungal suspensions were prepared in sterile saline (0.85% NaCl) and standardized to a 0.5 McFarland turbidity (1 × 10^6^ to 5 × 10^6^ CFU/mL) [66,67]. These suspensions were further diluted in RPMI 1640 medium to achieve a final concentration of 0.5 × 10^3^ to 2.5 × 10^3^ CFU/mL in each well.

#### 3.6.2. MIC and MFC Determination

The EO-BAI, nanoformulations, and pure constituents were dissolved in DMSO and subjected to two-fold serial dilutions in RPMI 1640, yielding a concentration range of 256–0.125 µg/mL. The final DMSO concentration was maintained below 1% (*v*/*v*) to avoid interference with fungal growth. Tests were conducted in 96-well microplates with a final volume of 200 µL per well (100 µL of inoculum and 100 µL of the respective treatment). Plates were incubated at 37 °C, and growth was monitored at 24 and 48 h by measuring absorbance at 540 nm using a microplate reader. The MIC_80_ was defined as the lowest concentration resulting in ≥80% growth inhibition compared to the untreated control [68]. Following CLSI guidelines, MIC and MFC values are reported as the specific concentrations obtained from the standardized two-fold dilution scale. Since this method focuses on discrete precision levels rather than continuous variables, standard deviations are not reported for these parameters, consistent with clinical interpretative criteria. To determine the MFC, 2 µL aliquots from wells showing no visible growth were subcultured onto SDA plates and incubated at 37 °C for 72 h. The MFC was identified as the lowest concentration that yielded no fungal growth, corresponding to a ≥99.5% reduction in the initial inoculum [69,70]. All assays were performed in triplicate across three independent experiments to ensure reproducibility.

#### 3.6.3. Time–Kill

Time–kill studies were performed according to standardized methodologies for antifungal fungicidal testing against yeasts, with minor adaptations [71,72]. *C. albicans* 10935 was grown on SDA at 35–37 °C for 24 h; then, 3–5 well-isolated colonies were suspended in sterile 0.85% NaCl and adjusted to a 0.5 McFarland standard (≈1–5 × 10^6^ CFU/mL). The suspension was then diluted in RPMI 1640 to obtain a final inoculum of approximately 5 × 10^5^ CFU/mL in each tube. Test tubes containing RPMI 1640 and the corresponding antifungal agent (EO, NE, or *α*-bisabolol) were inoculated and incubated at 35 °C under aerobic conditions with constant shaking. Samples were withdrawn at predetermined intervals (0, 3, 6, 9, 12, and 24 h), serially diluted in sterile saline, and plated onto SDA. After incubation for 24–48 h at 35 °C, colony counts were recorded and expressed as log_10_ CFU/mL. Fungicidal activity was defined as a ≥3 log_10_ CFU/mL reduction from the initial inoculum at 24 h, whereas smaller reductions were interpreted as fungistatic or inhibitory effects, in line with established criteria for antifungal testing. All experiments were performed in at least two independent biological replicates.

### 3.7. Molecular Docking

For the molecular docking study, the three-dimensional crystallographic structures of the N-terminal domain of Als9-2 from *C. albicans* in complex with the γ peptide of human fibrinogen (PDB ID: 2Y7L; resolution: 1.49 Å) [73], as well as the structure of sterol 14α-demethylase (CYP51) from *C. albicans* (PDB ID: 5TZ1; resolution: 2.0 Å) [53], were used as target proteins. Both structures were retrieved from the RCSB Protein Data Bank (https://www.rcsb.org/ (accessed on 17 January 2026)). Protein preparation was performed using AutoDock Tools v1.5.6. During this process, water molecules, metal ions, co-crystallized ligands, and any other molecules associated through non-covalent interactions were removed. Subsequently, Kollman charges and both polar and non-polar hydrogens were added, and the final files were exported in pdbqt format. The docking site was defined by setting the grid box center at −1.943 Å (X), −11.223 Å (Y), and 29.743 Å (Z) for 2Y7L, and at 67.837 Å (X), 36.744 Å (Y), and 39.072 Å (Z) for 5TZ1. For both targets, the grid box dimensions were set to 20 × 20 × 20 grid points along the X, Y, and Z axes. Docking simulations were carried out using 50 independent runs, with a maximum of 25,000,000 energy evaluations per ligand. Conformational clustering was performed using an RMSD cutoff of <1.6 Å. The resulting poses were ranked based on binding energy and cluster frequency. For further analysis, the pose showing the lowest binding energy and the most representative conformation within the corresponding cluster was selected. Ligand-receptor interactions were subsequently analyzed using Discovery Studio Visualizer v21.1.0.20298, which was also used to generate 2D and 3D interaction diagrams of the most stable selected conformation.

## 4. Conclusions

A stable nanoemulsion based on *H. foliosus* essential oil (NE-BAI) was developed, optimizing solubility and ensuring the sustained release of its active principles. The chemical profile, led by *α*-bisabolol, acts as a biological driver by interfering with the adhesion and membrane integrity of Candida, representing a robust alternative to azole resistance. The novelty of this work lies in the comprehensive characterization of NE-BAI efficacy and the elucidation of a mode of action linking sustained terpene delivery with the generation of oxidative stress (ROS) and the induction of apoptosis-like cell death, achieving potency comparable to fluconazole at 48 h. Although findings are currently limited to in vitro and in silico models, this study establishes the basis for future safety validations through hemolysis and cytotoxicity assays, as well as in vivo studies to confirm its clinical potential in complex biological environments.

## Figures and Tables

**Table 1:** Major components of EO-BAI according to GC-MS.

Nº	Components	Area ^a^ (%)	RI ^b^	RL ^c^	Identification
1	α-Terpinene	0.11	1023	1023	RL, MS, Co
2	*p*-Cymene	2.16	1030	1030	RL, MS, Co
3	Linalool	0.47	1101	1102	RL, MS, Co
4	Fenchol	0.21	1122	1224	RL, MS
5	*trans*-2-Menthenol	0.80	1129	1130	RL, MS, Co
6	*cis*-2-Menthenol	0.89	1148	1148	RL, MS
7	endo-Borneol	0.83	1177	1178	RL, MS, Co
8	terpinen-4-ol	27.27	1188	1188	RL, MS, Co
9	*p*-Cymen-8-ol	0.65	1193	1194	RL, MS, Co
10	α-Terpineol	2.21	1199	1200	RL, MS, Co
11	*cis*-Piperitol	0.18	1204	1204	RL, MS
12	*trans*-Piperitol	0.42	1216	1216	RL, MS
13	Geraniol	0.84	1258	1258	RL, MS, Co
14	γ-Terpinene	1.09	1269	1269	RL, MS
15	*trans*-Ascaridol glycol	0.18	1282	1282	RL, MS
16	Cuminol	0.69	1294	1294	RL, MS
17	Geranyl formate	0.29	1305	1305	RL, MS
18	Carvacrol	0.58	1309	1310	RL, MS, Co
19	Eugenol	1.12	1322	1322	RL, MS, Co
20	Geranyl acetate	0.95	1383	1384	RL, MS, Co
21	Copaene	0.46	1389	1390	RL, MS
22	Methyleugenol	1.33	1408	1408	RL, MS, Co
23	*trans*-α-Bergamotene	0.44	1437	1437	RL, MS
24	Caryophyllene	1.11	1448	1448	RL, MS, Co
25	Alloaromadendrene	0.58	1458	1458	RL, MS
26	Selina-5,11-diene	0.13	1462	1462	RL, MS
27	Eudesma-1,4(15),11-triene	2.42	1478	1478	RL, MS
28	γ-Muurolene	0.77	1481	1481	RL, MS
29	Germacrene D	1.18	1491	1491	RL, MS
30	(1*S*,2*S*,4*S*)-trihydroxy-*p*-menthane	0.53	1497	1497	RL, MS
31	β-Selinene	1.04	1506	1506	RL, MS
32	α-Selinene	2.17	1515	1516	RL, MS
33	α-Farnesene	0.24	1524	1524	RL, MS
34	γ-Cadinene	1.10	1531	1531	RL, MS
35	Cadina-1(10),4-dien-8α-ol	2.32	1538	1538	RL, MS
36	Kessane	0.60	1551	1550	RL, MS
37	Caryophyllene oxide	2.02	1561	1561	RL, MS, Co
38	Globulol	0.71	1582	1582	RL, MS
39	Germacrene-4-ol	0.33	1586	1586	RL, MS
40	Spathulenol	6.70	1600	1600	RL, MS
41	Isoaromadendrene epoxide	2.39	1608	1608	RL, MS
42	Tetradecanal	1.48	1612	1612	RL, MS
43	*epi-α*-cadinol	0.54	1634	1634	RL, MS
44	Muurola-4,10(14)-dien-1β-ol	3.77	1649	1650	RL, MS
45	γ-Eudesmol	0.88	1654	1654	RL, MS
46	β-Eudesmol	1.66	1657	1658	RL, MS
47	τ-Cadinol	1.45	1661	1662	RL, MS
48	α-Eudesmol	4.95	1676	1676	RL, MS
49	*α*-Bisabolol	10.40	1699	1699	RL, MS, Co
50	t-Muurolol	0.40	1725	1726	RL, MS
51	Tetradecanoic acid	0.23	1763	1762	RL, MS
52	10-*epi*-γ-eudesmol	0.22	1773	1773	RL, MS
53	Nootkatone	0.35	1814	1814	RL, MS, Co
54	Hexadecanol	0.27	2120	2218	RL, MS
55	Tetracosane	0.79	2294	2294	RL, MS
56	Pentacosane	0.15	2494	2484	RL, MS
	Total identified	98.05			
	Hydrocarbon monoterpenes	3.36			
	Oxygenated monoterpenes	36.17			
	Monoterpene esters	1.24			
	Hydrocarbon sesquiterpenes	11.64			
	Oxygenated sesquiterpenes	39.69			
	Phenols	0.58			
	Phenylpropanoids	2.45			
	Hydrocarbon alkane	0.94			
	Fatty alcohol	0.27			
	Fatty aldehyde	1.48			
	Fatty acid	0.23			

^a^ Area: surface area of GC peak; ^b^ RI: experimental retention index for non-polar column; ^c^ RL: bibliographic retention index for non-polar column; MS: mass spectra; Co: co-elution with standard compounds available in our laboratory.

**Table 2:** Stability parameters for NE-BAI and vehicle control (VC).

Sample	Ps (nm)	PDI	ZP (mV)	pH	EE (%)
NE-BAI	21.80 ± 5.1	0.583 ± 0.18	−3.47 ± 0.3	6.90	88.72
VC	23.40 ± 5.4	0.575 ± 0.21	−3.76 ± 0.4	6.88	0

Ps: Particle size; PDI: Polydispersity index; ZP: Zeta Potential; EE: Encapsulation efficiency.

**Table 3:** Antifungal activity of treatments against *Candida* strains at 48 h.

	*C. albicans*			*C. glabrata*			*C. lusitaniae*			*C. tropicalis*		
Sample	MIC	MFC	R	MIC	MFC	R	MIC	MFC	R	MIC	MFC	R
EO-BAI	32	64	2	128	256	2	8	32	4	32	64	2
NE-BAI	64	128	2	256	>256	-	16	64	4	64	128	2
VC	I			I			I			I		
*α*-bisabolol	16	32	2	32	64	2	2	8	4	8	16	2
terpinen-4-ol	256	>256	-	128	256	2	32	32	1	64	128	2
Fluconazole	64	128	2	64	64	1	32	64	2	16	16	1
Itraconazole	16	16	1	2	4	2	4	16	4	1	2	2
Clotrimazole	64	128	2	256	256	1	>256	>256	-	>256	>256	-
DMSO	I			I			I			I		

All values are expressed in µg/mL. MIC: Minimum inhibitory concentration (MIC_80_); MFC: Minimum fungicidal concentration; R: MFC/MIC ratio; -: not applicable; I: inactive.

**Table 4:** Time–kill kinetics of treatments against *C. albicans* over 24 h.

Treatment	0 h	3 h	6 h	9 h	12 h	24 h
C0	7.37	7.15	6.64	6.92	7.33	7.03
VC	7.05	6.88	6.71	6.83	7.26	7.27
EO-BAI 1	7.04	6.49	6.70	6.97	7.55	7.45
EO-BAI 4	6.39	6.13	6.42	6.62	6.52	7.20
*α*-bisabolol 1	7.11	6.62	7.01	6.53	7.34	7.26
*α*-bisabolol 4	6.11	4.98	6.18	6.33	6.56	5.83
NE-BAI 1	7.15	6.52	6.67	6.60	7.34	7.48
NE-BAI 4	6.76	6.62	6.94	6.57	7.31	7.43

Data are expressed as log_10_ CFU/mL. C0: Growth control; VC: Nanoemulsion vehicle; EO-BAI, *α*-bisabolol, and NE-BAI tested at two concentrations: (1) 1×MIC and (4) 4×MIC.

**Table 5:** Binding energies and key interactions for major compounds from EO-BAI against *C. albicans* Als9-2 (PDB: 2Y7L) and CYP51 (PDB: 5TZ1) targets.

Compound	Enzyme (PDB ID)	Binding Energy (kcal/mol)	Key Interactions
Terpinen-4-ol	2Y7L	−4.56	Val22;Arg171;Tyr297;Pro29;Val19;Leu179;Trp294
	5TZ1	−4.28	Ser378;Leu376;Tyr118;Leu121;Pro230;Phe380
*α*-bisabolol	2Y7L	−5.98	Phe225;Tyr23;Trp294;Ser170;Pro29; Val22; Leu179;Val19
	5TZ1	−6.01	Tyr118;Leu121;Tyr132;Leu376;Pro230;Phe233; Phe380;Met508
Fluconazole	2Y7L	−7.97	Ser170;Trp295;Glu27;Gly296;Ile172;Leu179;Pro29;Arg171;Val22;Trp294
	5TZ1	−6.91	Leu121;Pro230;Met508;Tyr64;Pro375;Leu376;Hse377
Itraconazole	2Y7L	−9.96	Tyr21;Val22;Arg171;Ile167;Val161;Ser169;Trp223;Thr168;Tyr23;Asn224;Ser159
	5TZ1	−10.51	Ala61;Phe58;Leu87;Leu121;Leu376;Ile304;Pro230;Ile471;Met508;Pro462;Gly472
Clotrimazole	2Y7L	−6.81	Val22;Trp294;Val101;Tyr21;Tyr23
	5TZ1	−9.01	Leu139;Leu471; Cys470;Tyr132;Lys143;Hsd468

## Data Availability

The original contributions presented in the study are included in the article and Supplementary Material, further inquiries can be directed to the corresponding author.

## Supplementary Materials

The following supporting information can be downloaded at: https://www.mdpi.com/article/10.3390/ph19050759/s1, Table S1: Minimum inhibitory concentration (MIC_80_ µg/mL) values against *Candida* strains at 24 h of incubation.
