# Supplementary material for: Candicidal Effect of a Nanoemulsion Based on the Essential Oil of the Medicinal Plant Haplopappus foliosus: Role of Main Compounds in Yeast Inhibition
Source: Pharmaceuticals (Basel). 2026 May 12;19(5):759. doi: 10.3390/ph19050759 (PMC13210062; doi:10.3390/ph19050759)
Supplement: Supplementary file 1 [file pharmaceuticals-19-00759-s001.zip › pharmaceuticals-4294460-supplementary.pdf]

Candicidal Effect of a Nanoemulsion Based on the Essential Oil of the Medicinal Plant *Haplopappus foliosus*: Role of Main Compounds in Yeast Inhibition

Alejandro Madrid[1*], Bastián Fuentes[2,3], Camila Araneda[4], Iván Montenegro[3] Valentina Silva[1] and Evelyn Muñoz[5]

[1] Laboratorio de Productos Naturales y Síntesis Orgánica (LPNSO), Facultad de Ciencias Naturales y Exactas, Universidad de Playa Ancha, Leopoldo Carvallo 270, Valparaíso; alejandro.madrid@upla.cl; silvapedrerosv@gmail.com.
[2] Doctorado en Ciencias Mención Biofísica y Biología Computacional, Universidad de Valparaíso, Chile; bastian.fuentes@uv.cl.
[3] Center of Interdisciplinary Biomedical and Engineering Research for Health (MEDING), Escuela de Obstetricia y Puericultura, Facultad de Medicina, Universidad de Valparaíso, Angamos 655, Reñaca, Viña del Mar 2520000, Chile; bastianfuentes.valdes@gmail.com; ivan.montenegro@uv.cl
[4] Instituto de Química, Pontificia Universidad Católica de Valparaíso, Chile; camila.araneda.v@mail.pucv.cl
[5] Centro de Investigación Austral Biotech, Facultad de Ciencias, Universidad Santo Tomás, Avda. Ejercito 146, Santiago 8320000, Chile; nelsoncarofu@santotomas.cl
[6] Departamento de Química Orgánica, Facultad de Ciencias Químicas, Universidad de Concepción, Concepción, Chile; emunoznu@udec.cl

* Correspondence: alejandro.madrid@upla.cl; Tel.: +56-32-2205098

Table S1. Minimum inhibitory concentration (MIC$_{80}$ μg/mL) values against *Candida* strains at 24 h of incubation.

Treatment	*C. albicans*	*C. glabrata*	*C. lusitaniae*	*C. tropicalis*
**EO-BAI**	32	128	8	32
**NE-BAI**	>256	>256	32	128
***α*-bisabolol**	8	32	2	8
**4-terpineol**	32	128	32	64

All values are expressed in μg/mL.
